# Physicochemical properties and sensory attributes of cookies prepared from sorghum and millet composite flour

**DOI:** 10.1002/fsn3.2942

**Published:** 2022-05-28

**Authors:** Moneera O. Aljobair

**Affiliations:** ^1^ Nutrition and Food Science (PHD), Department of Physical Sport Science, College of Education Princess Nourah bint Abdulrahman University Riyadh Saudi Arabia

**Keywords:** bioactive properties, composite flour, cookies, millet, sorghum

## Abstract

Sorghum and millet are rich in nutrients and bioactive compounds. They can enhance nutrition and health. This study was aimed to develop sorghum–millet gluten‐free cookies from sorghum and millet composite flour. Cookies were made using 100% wheat WF100%, sorghum SF100% ,and millet MF100% flours and composite flours of 25% sorghum–75% millet (SF25%–MF75%), 50% sorghum−50% millet (SF50%–MF50%), and 75% sorghum–25% millet (SF75%–MF25%). The physicochemical, nutritional, bioactive, and sensory quality attributes of the developed cookies were evaluated.

The results of flours displayed that the highest values of peak viscosity and final viscosity were observed in SF100%, breakdown and pasting temperature were observed in WF100%, setback viscosity in composite flour of SF25%–MF75%, and hardness and chewiness in WF 100%. Regarding cookies analysis results, the highest values of hardness were shown in WF100% and SF100% cookies, and the highest water activity was observed in the cookies of (SF25%–MF75% and SF50%–MF50%), and the (SF75%–MF25%) cookies had the best diameter and thickness. The lowest water‐holding capacity (WHC) and oil‐holding capacity (OHC) were found in (WF100%) cookies, while they were high in the cookies of SF and MF flours and their combinations (*p* ≤ .05). The cookies made using the composite flour of SF25%–MF75% appeared to have the uppermost values of moisture, protein, fat, total phenolic content (TPC), and antioxidant activity. They have the highest contents (*p* ≤ .05) of potassium, sodium, magnesium, and calcium. The scores of all sensory attributes of all cookie types were higher than the cutoff score (5). Overall, the incorporation of sorghum and millet flour in cookies formulation enhanced the physicochemical, nutritional, and bioactive properties without major effect on the sensory attributes of the product, which make it excellent nutritive and healthy sorghum–millet gluten‐free cookies.

## Graphical abstract

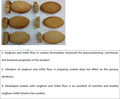

## 1 INTRODUCTION

Sorghum and millet are important sources of protein and energy for many people in arid and semiarid regions (ICRISAT/FAO, 1996). These cereal grains are rich in nutrients such as proteins, minerals, bioactive compounds, and cholesterol‐lowering waxes (Taylor et al., 2006). They are also considered as the best food crops for future human use due to their high tolerance to drought and hot temperature and their little input requirements during growth (Taylor et al., 2006). These vital cereal grains are used for the preparation of various foods and beverages in developing countries; however, they are still majorly used as animal feed in developed countries (Mohamed et al., 2019; Taylor et al., 2006). Due to global warming, water shortage, population growth, and health concerns about gluten‐containing foods, the popularity and importance of sorghum and millet products are greatly increased in recent decades. Gluten‐free foods are highly required for the treatment of celiac disease, an immune‐mediated enteropathy activated by the consumption of gluten‐containing foods of wheat, barley, and rye origins (Brites et al., 2018). In recent years, the production and consumption of snacks are highly increased due to urbanization, speedy work, and fast food, and increased numbers of working women (Rao et al., 2018). Among snacks, cookies are considered an important food as they have high acceptability, a wide range of crispiness, digestibility, shapes, flavors, and tastes, low cost, and long shelf‐life (Sudha et al., 2007). The development of gluten‐free bakery products, such as bread, pastas, noodles, cookies, pizza, and biscuits from sorghum and millet, represents a major challenge for the food industry because the absence of gluten affects the structure of the developed products (Torbica et al., 2012). Currently, gluten‐free bakery products deliver lower protein, fiber, and mineral content and elevate glycemic index (GI) than gluten‐containing foods. The currently low mineral content can be increased with the use of mineral‐rich ingredients such as amaranth, buckwheat, or flaxseed flour (Sophie & Luca, 2018).

Gluten‐free cookies from sorghum and millet could be nutritious and healthy due to their richness of chemical constituents (dietary fibers, proteins, fatty acids, and minerals) and bioactive compounds (Brites et al., 2018; Taylor et al., 2006; Torbica et al., 2012).

Due to the high content of micronutrients and dietary fiber, in addition to the low GI and the lack of gelatin, the development of gelatin‐free products from corn and millet is considered a necessity for the prevention of diseases related to gelatin, diabetes, obesity, and other diseases related to high glycemic factors. They also contain significant amounts of phytochemicals that make them a therapeutic food for the prevention and treatment of many chronic diseases. Accordingly, this study aimed to develop cookies using sorghum and millet flour and their blends and to evaluate the physicochemical, nutritional, bioactive, and sensory quality attributes of the developed cookies.

## 2 MATERIALS AND METHODS

### 2.1 Materials

#### 2.1.1 Row materials

All ingredients (sorghum and millet flour, wheat flour, refined sugar, guar gum, shortening, baking powder, cinnamon powder, salt, and eggs) used to prepare cookies were purchased from local markets in Riyadh, Saudi Arabia.

#### 2.1.2 Chemicals and reagents

The chemicals used such as (HCl, HNO3, H_2_O_2_, AlCl_3_, NaCO_3_, gallic acid, and 2,2‐diphenyl‐1‐picrylhydrazyl [DPPH]). They were of analytical grade and were obtained from Sigma (Sigma‐Aldrich).

### 2.2 Water‐ and oil‐holding capacity

The water‐holding (WHC) and oil‐holding capacity (OHC) for all flour samples were assessed following the method described by Berton et al. (2002) with some modifications. The method was based on the AACC method no. 88–04 (AACC, 2000). Briefly, 1.0 g from the flour samples (W0) was dissolved in 5 ml of water or oil and vortexed for 10 s. The sample was left standing for 30 min at room temperature (25 ± 2°C), then subjected to centrifugation at 2000 × g for 10 min, and the sediment was weighed (W1). The WHC or OHC was calculated as the grams of water or oil absorbed per gram of flour samples, according to the following equation: WHC or WOH (g/g) = W1‐W0/W0.

Where: W0 represents the initial weight (g) of samples and W1 represents the final weight (g) of samples. The results are expressed as the mean values of three replicates.

### 2.3 Pasting properties of composite flour dispersions

The wheat flour and the blends of composite (sorghum and millet) flour with 2% guar gum were prepared. The measurement of pasting properties for wheat and composite flours was done by using the Rapid Visco Analyzer (RVA) (Newport Scientific). The moisture content of the samples was adjusted to 14%, and then 3.5 g of each sample was weighed into aluminum canisters, and then distilled water was added to adjust the weight to 28 g. The sample was mixed, and the obtained slurry was poured into an aluminum pan, loaded into the RVA chamber, and held for 50 s at 50°C. After that, the temperature was raised to 95°C at 13.15°C/min in 3.42 min and then kept for 3.30 min at this temperature. The samples were subjected to cooling to 50°C at a rate of 12.93°C/min in 3.48 min and then held for 2 min at this temperature. The samples were measured in triplicate, and the data were processed by using Thermocline window software. Peak viscosity (PV), final viscosity (FV), peak time (Pt), peak temperature (PT), setback (SB), and breakdown (BD) were the main parameters measured.

### 2.4 Gel texture analysis

After the RVA tests, the gel was subjected to overnight storage at 25°C in a beaker (25 ml volume) with the height and internal diameter of 35 mm and 30 mm, respectively. A Brookfield CT3 Texture Analyzer (Brookfield Engineering Laboratories, Inc.) was used for texture analysis. In the analyzer, the gel was compressed at a speed of 0.5 mm/s using two penetration cycles to a distance of 10 mm using 12.7 mm (width) × 35 mm (length) cylindrical probe. Thereafter, the texture parameters of the gels such as hardness, cohesiveness, adhesiveness, and springiness were measured. The harness and cohesiveness results were used to calculate the gumminess as reported previously (Hussain et al., 2013).

### 2.5 Cookies preparation

The preparation of cookies was done according to the AACC method no. 10–50 (AACC, 2000), with slight modifications. The ingredients used for cookies are mentioned in Table 1. Five flour samples (wheat, sorghum, millet, sorghum 25% with 75%millet, sorghum 50% with millet 50%, and sorghum 75% with 25%) with 2% addition of gum for each flour except the wheat flour. To the mixing bowl, the materials were added in a specific order. Sugar, egg, and shortening were initially added to the bowl and then subjected to 2 min mixing at a low speed. Perfectly mixed flour sample, baking powder, cinnamon powder, and salt were added and subjected to 1 min mixing at low speed. In the end, dextrose and distilled water were added and continually mixed until homogenization. After that, the rolling pins were used to shape the dough into cookie sheets of 5‐mm thickness. Then, the sheeted cookies were sliced into cookie shapes, placed on baking trays, and subjected to the baking process (225°C for 8 min) in a cooking oven. After cooling to room temperature, the cookies were sealed in plastic bags and stored at room temperature for further analysis.

**TABLE 1:** Cookies ingredients

	WF100%	SF100%	MF100%	SF25%–MF75	SF50%–MF50%	SF75%–MF25%
Wheat flour(g)	200	0	0	0	0	0
Sorghum flour(g)	0	200	0	50	100	150
Millet flour(g)	0	0	200	150	100	50
Guar gum(g)	4	4	4	4	4	4
Shortening(g)	55	55	55	55	55	55
Baking powder(g)	2	2	2	2	2	2
Salt(g)	2	2	2	2	2	2
Cinnamon	4	4	4	4	4	4
Sugar(g)	110	110	110	110	110	110
Egg(g)	50	50	50	50	50	50
Dextrose 6% (ml)	30	30	30	30	30	30
Distilled water (ml)	14	14	14	14	14	14

### 2.6 Cookie quality analysis

#### 2.6.1 Cookie physical analysis

The diameter of cookies, thickness, and spread ratio were assessed as described previously (Kaur et al., 2015). Briefly, a digital Vernier caliper was used to measure the average thickness (mm) of six cookies. The same for diameter, six cookies were lined edge to edge, and the average width (mm) was calculated by rotating the cookies at 90 degree angle. The ratio between the thickness and the diameter was considered as the spread factor of cookies.

#### 2.6.2 Water activity measurement

AquaLab Series 3 Water activity meter (Decagon Devices) was applied for the determination of water activity of cookies water activity (aw) at 25°C (Inglett et al., 2015).

#### 2.6.3 Cookie color analysis

Cookie color was measured at room temperature in terms of L* (lightness), a* (redness), and b* (yellowness) values in triplicate using a color grader (Satake, NCGA, Japan) with a D65 light source.

#### 2.6.4 Cookie texture analysis

A TA‐XT plus texture analyzer (Stable Micro Systems) was used to measure the texture profile of the cookie samples as described previously (Gül et al., 2018).

### 2.7 Chemical analysis

#### 2.7.1 Proximate composition of cookies

Cookies moisture, crude fat, crude protein, and ash were determined according to the (AOAC, 2003). Total carbohydrates were calculated by the difference method.

#### 2.7.2 Minerals’ analysis

The minerals were evaluated in cookies samples after acid digestion of the samples. Briefly, to 0.5 g of samples in a Teflon tube 1.6 ml of 37% HCl and 1.7 ml of 65% HNO3 were mixed with the sample, and after standing for 30 min at room temperature (RT), 1.7% of H2O2 (30%) was added and mixed well. The digestion proceeded with heating in a microwave, a temperature ramp‐up to 170°C for 20 min, and then it was sustained at the same temperature for 15 min. The power was set in the range of 290–1800 W, and at the end of the program, the samples were cooled to room temperature, and the solution was transferred to 25.0 ml conical tubes and filled with Milli‐Q water. The mineral contents were measured using a PerkinElmer Optima 7300DV ICP‐OES (inductively coupled plasma optical emission spectrometry) (Waltham, USA) under the following conditions: measuring power 1300 W; integration time of signal 1 s; plasma gas flow 15 L min‐1; auxiliary gas flow 1.5 L min‐1; nebulization gas flow 0.70 L min‐1; and pumping rate of the sample 0.70 ml min‐1.

### 2.8 Total phenols and radical scavenging activity determination

Initially, 1 g of cookies was extracted in ethanol (25 ml) in a shaker for 24 h and then centrifuged for 15 min at 10,000 × g. The mixture was filtered by using filter paper (Whatman #41), and the transparent supernatant was separated. The volume of the collected supernatant was readjusted to 25 ml and kept at 4°C for the estimation of phenolic and radical scavenging activity. The cookie extract was estimated for total phenolic content following the method described by Wu et al. (2007) with minor modifications. A gallic acid standard curve was prepared for comparison, and results were presented as equivalents of gallic acid per unit weight (g) of cookies. For the measurement of radical scavenging activity, the DPPH method was adopted (Akillioglu & Karakaya, 2010).

### 2.9 Sensory evaluation of cookies

The panel was composed of 20 semitrained panelists of the male students and staff of the Department of Food Science and Nutrition, King Saud University. The cookie samples were coded using a three‐digit number and randomly presented to the panelists. The sensory attributes (color, taste, aftertaste, texture, appearance, aroma, and overall acceptability) were assessed using a 9‐point hedonic scale (1 = dislike extremely, 5 = neither dislike nor like, and 9 = like extremely) (Gat & Ananthanarayan, 2016).

### 2.10 Statistical analysis

All data were collected in three replicates at least and analyzed by using the one‐way analysis of variance (ANOVA) and Duncan’s Multiple Range (DMR) test at sig ≤0.05 was used to compare means using SPSS (IBM Statistical Analysis Version 21).

## 3 RESULTS AND DISCUSSION

### 3.1 Pasting properties and texture profile of composite flour

The results showed significant (*p* ≤ .05) differences in the pasting properties and texture of wheat flour (WF), sorghum flour (SF), millet flour (MF), and composite flour of different levels of sorghum and millet flours (Table 2). The highest values of peak viscosity (PV) and final viscosity (FV) were found in SF100%, and the least values were seen in composite flour of SF50%–MF50% and WF100%, respectively (*p* ≤ .05). Increasing millet flour in the blend resulted in a reduction of peak and final viscosities, which are likely due to the differences in the nature of starch between millet and sorghum flours and diluting effects of millet starch on the sorghum starch in the blend. Similarly, Hussain et al. (2020) reported that increasing the millet flour reduced the peak and final viscosities of the wheat–millet composite flour. In addition, Chauhan et al. (2015) observed a reduction in the peak viscosity following the inclusion of nonwheat flour in the blends. The highest values of breakdown and pasting temperature were noted in control (WF100%), while the minimum values were found in composite flour of SF50%–MF50% and MF100%, respectively (*p* ≤ .05). It is because of the presence of gelatin in wheat flour and its absence in sorghum and millet flours. The highest setback viscosity value was observed in composite flour of SF25%–MF75%, and the least was seen in SF50%–MF50% composite flour (*p* ≤ .05), indicating that the inclusion of MF in the composite flour increases the setback viscosity because of amylose retrogradation tendency in this flour mixture (Hussain et al., 2020). Based on the results of this study, the differences in the setback viscosity of the composite flours can be attributed to the variation in the amylose contents and granule size of two different starches (millet and sorghum) (Hussain et al., 2020; Shafi et al., 2016). The findings of this study indicate that the incorporation of a high percentage of MF in the formulation modifies the pasting properties of the blend.

**TABLE 2:** Pasting properties and textural profile analysis (TPA) of composite flour

Properties	Control (WF 100%)	SF (100%)	MF (100%)	SF25%–MF75%	SF50%–MF50%	SF75%–MF25%
Pasting properties						
Peak viscosity (cP*)	1225 ± 27.8b	1333.66 ± 40.7a	1059 ± 24.8c	1066 ± 58.02c	959 ± 69.3d	1177.33 ± 45.5b
Breakdown (cP)	512 ± 10.8a	122.33 ± 9.1c	149.66 ± 4.5b	147.66 ± 4.3b	94.66 ± 4.5d	146.66 ± 5.8b
Final viscosity (cP)	1526 ± 6.8f	1944.33 ± 6.5a	1643.66 ± 6.2d	1772.66 ± 4.5c	1563 ± 6.6e	1813 ± 11.7b
Setback (cP)	786.66 ± 5.8b	747 ± 12.1d	733 ± 8.8e	819 ± 6.8a	716.66 ± 6.5f	765.66 ± 5.1c
Pasting temp. (°C)	86.413 ± 0.15a	83.22 ± 0.05c	80.70 ± 0.01e	81.58 ± 0.02d	84.86 ± 0.03b	81.65 ± 0.04d
Textural profile analysis						
Hardness (g)	42 ± 1.0a	29 ± 1.0b	9.0 ± 1.0e	9.33 ± 1.5e	11.66 ± 1.1d	22.33 ± 0.5c
Cohesiveness	0.42 ± 0.02c	0.46 ± 0.02c	0.59 ± 0.03b	0.75 ± 0.03a	0.37 ± 0.02d	0.46 ± 0.01c
Springiness (mm)	8.53 ± 0.3c	9.66 ± 0.3c	9.26 ± 0.1b	6.30 ± 0.1d	8.40 ± 0.2c	10.80 ± 0.7a
Adhesiveness	0.53 ± 0.1a	0.56 ± 0.05a	0.13 ± 0.05b	0.16 ± 0.05b	0.53 ± 0.5a	0.43 ± 0.05a
Chewiness (g)	152.17 ± 15.1a	128.91 ± 8.9b	49.67 ± 7.8c	44.33 ± 7.9c	36.90 ± 2.6c	112.24 ± 3.1b

*Note*: Values followed by different letters within each row significantly different (*p* ≤ .05).

Abbreviations: *cP, Centipoise; MF, Millet flour; SF, Sorghum flour; WF, Wheat Flour.

The texture profile of the composite flour paste was also affected by the formulation ingredients (*p* ≤ .05). The highest values of hardness were found in control (100% WF), and the last ones were seen in MF100% and composite flour with a high level of MF (SF25%–MF75%), suggesting the negative influence of MF on the hardness of the dough. However, it increased the cohesiveness when composted with sorghum at the aforementioned ratio (SF25%–MF75%). The chewiness value was the highest in control (100% WF), followed by sorghum flour (SF100%), and the least in the composite of SF50%–MF50%. The reduction of hardness following the addition of MF in this study could be attributed to the low starch, amylose, and amylopectin content of millet flour, compared to wheat and sorghum flour. The gel hardness is referred to as the retrogradation of the starch in the flour (Hussain et al., 2020). The variation in the content and composition among the millet, wheat, and sorghum flour likely affected the textural profile of the obtained gels of the blends. In agreement with the above findings, Hussain et al. (2020) reported high hardness and chewiness in the gel formed using wheat flour, and the addition of millet flour significantly reduced the hardness of the blend. In addition, Joshi et al. (2014) stated that reducing the starch fraction significantly reduced the hardness of the flour blends. Overall, the incorporation of millet and sorghum flours affects the texture profile of the composite flour.

### 3.2 Physical, textural, functional, and color properties of cookies

**FIGURE 1:**
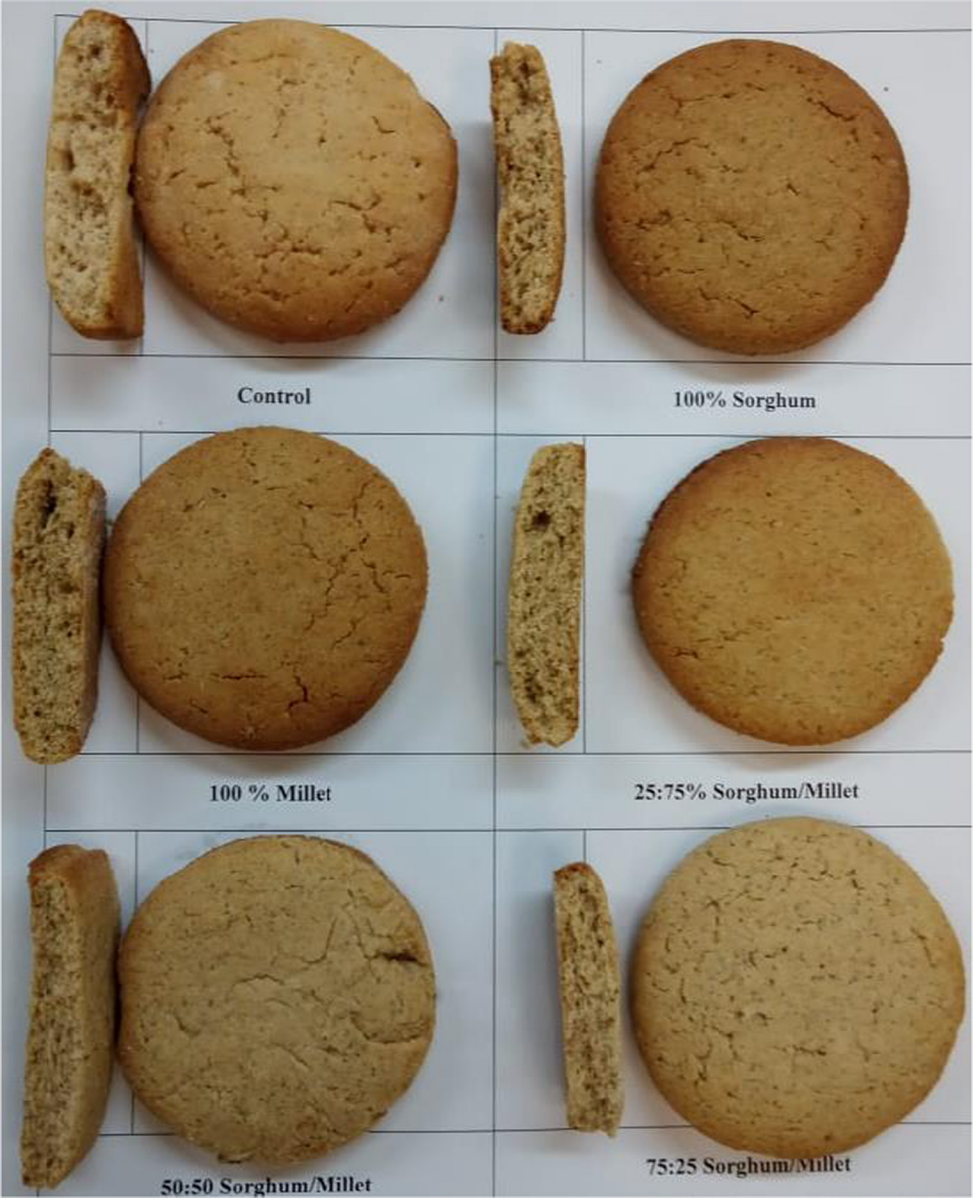
Photograph of cookies

Significant variations (*p* ≤ .05) in the physical, textural, functional, and color properties of cookies prepared using WF, SF, MF, and composite flour of different levels of sorghum and millet flours were observed (Table 3, Figure 1). The hardness was high in cookies made from WF100%, SF100%, and composite flour with a high level of SF, and it was low in cookies made of composite flour containing high levels of MF (*p* ≤ .05), these results’ findings suggesting that the incorporation of high level of millet in the composite flour reduces the hardness of the product. The fracturability was low in cookies made using MF100%. It was high in other samples, whereas it was insignificant among them. Water activity was high in cookies made using composite flour (SF25%–MF75% and SF50%–MF50%) and low in cookies made using SF100% and composite flour of SF75%–MF25% (*p* ≤ .05). The concomitant reduction of the hardness and increment of the fracturability following the incorporation of MF in the cookies that developed in this work are likely due to the dilution of gluten and the existence of higher fiber in the blends with higher levels of MF. In agreement with our findings, numerous reports have shown that the addition of different gluten‐free flours to cookie formulations concomitantly increased the fracturability and reduced the hardness of the cookies (Chauhan et al., 2015; Hussain et al., 2020; Jan et al., 2015).

**TABLE 3:** Physical, textural, functional, and color properties of cookies

Properties	Control (WF 100%)	SF (100%)	MF (100%)	SF25%–MF75%	SF50%–MF50%	SF75%–MF25%
Textural properties						
Hardness (*N*)	91.90 ± 11.1a	90.20 ± 6.8a	65.28 ± 0.99b	49.10 ± 1.7c	56.93b ± 0.3c	86.03 ± 9.1a
Fracturability (mm)	6.27 ± 0.6a	5.58 ± 0.5a	4.13 ± 0.1b	5.98 ± 0.6a	60.02 ± 0.2a	5.42 ± 0.2a
Water activity (aw)	0.47 ± 0.0b	0.35 ± 0.0d	0.39 ± 0.0c	0.45 ± 0.01a	0.45 ± 0.04a	0.36 ± 0.02d
Physical properties						
Diameter (mm)	57.67 ± 1.60c	59.77 ± 0.90abc	58.87 ± 2.10bc	60.15 ± 1.10ab	60.05 ± 0.90ab	61.97 ± 1.40a
Thickness (mm)	10.40 ± 1.80c	10.87 ± 0.60ab	11.12 ± 1.20ab	11.12 ± 0.6ab	12.12 ± 0.30a	12.45 ± 1.20a
Spread ratio	5.17 ± 0.90ab	5.51 ± 0.30ab	5.32 ± 0.40ab	5.95 ± 0.30a	5.33 ± 0.10ab	5.00 ± 0.40b
Functional properties						
WHC	1.72 ± 0.01b	2.21 ± 0.04a	2.16 ± 0.10a	2.14 ± 0.04a	2.16 ± 0.06a	2.25 ± 0.05a
OHC	1.75 ± 0.03b	1.80 ± 0.10ab	1.78 ± 0.02ab	1.75 ± 0.04b	1.81 ± 0.03ab	1.88 ± 0.03a
Color attributes						
L*	76.55 ± 0.6a	69.02 ± 0.3d	63.55 ± 0.3e	69.22 ± 0.4d	71.77 ± 0.1c	74.32 ± 0.1b
a*	1.74 ± 0.5b	3.40 ± 0.0a	3.25 ± 0.1a	0.89 ± 0.0c	−1.46 ± 0.1d	−2.48 ± 0.5e
b*	37.55 ± 0.0a	35.46 ± 0.1b	34.30 ± 0.1c	32.56 ± 0.1d	29.50 ± 0.2e	26.65 ± 0.3f

*Note*: Values followed by different letters within each row significantly different (*p* ≤ .05).

Abbreviations: a*, Redness; b*, Yellowness; L*, Lightness; MF, Millet flour; SF, Sorghum flour; WF, Wheat flour.

Regarding the physical properties of the cookies, insignificant differences were observed between the samples with a generally high level of diameter and thickness of the cookies made using an increased percentage of SF in the composite flour (SF75%–MF25%) and least values in cookies made using WF100% (Table 3). The spread ratio was high in cookies made using a high level of MF in the composite flour (SF25%–MF75%). The high diameter and thickness of the cookies made using a high level of SF in the blend are likely due to the high fiber content of the SF compared to that made using fine wheat flour. The increased spread ratio of cookies made using a high level of MF could be attributed to the high oil content in the flour as the whole flour was used, which contained the germ in addition to a high particle size of the whole sorghum and millet flour compared to fine wheat flour (Belorio et al., 2019; Paesani et al., 2020).

The water‐holding capacity (WHC) and oil‐holding capacity (OHC) were low in the cookies made using control flour (WF100%), and they were high in cookies made using SF and MF flour separately or in combinations (*p* ≤ .05). The values of these functional properties were insignificantly high in cookies made using high levels of SF (SF75%–MF25%) in the formulations suggesting the influence of SF on these properties. High WHC and OHC in cookies made using SF or MF and their combination could be attributed to the use of whole flour of sorghum and millet, which contain the bran that is rich in fiber and protein, and which are known to bind more water and oil in their structure (Paesani et al., 2020; Sharma et al., 2016).

The color attributes of cookies made using WF, SF, MF, and composite flour of different levels of sorghum and millet flours showed significant (*p* ≤ .05) differences (Table 3, Figure 1). The highest value of lightness (L*) was found in the cookies made using WF100%, and the least value was seen in those made using MF100% (*p* ≤ .05). The highest value of redness (a*) was found in the cookies made from sorghum (SF100%) and millet (MF100%) flours, whereas the least values were found in cookies made from the composite flour of SF75%–MF25% (*p* ≤ .05). The yellowness (b*) values were high in cookies made using wheat flour, followed by those of sorghum and millet flours, while the least values were seen in cookies made using SF75%–MF25% composite flour (*p* ≤ .05). The reduction of lightness and yellowness and increase in the redness of cookies made from SF and MF and their combinations are likely due to the whole flours of MF and SF used, which contain the constituents of the bran with darker colors such as fibers, phenolics, and flavonoids (Brites et al., 2018; Paesani et al., 2020; Torbica et al., 2012). In addition, Millard reaction during the baking process could also contribute to the color changes of cookies (Giuberti et al., 2018).

### 3.3 Chemical composition and bioactive properties of cookies

The results of the approximate composition, bioactive properties, and mineral contents of cookies made using different flour types are shown in Table 4. The highest values of moisture, protein, and fat were found in cookies made using composite flour of SF25%–MF75%, whereas the least values of moisture and protein were seen in cookies made using increased levels of SF in the composite flour (SF75%–MF25%), and the least value of fat content was seen in those made using pure sorghum flour (SF100%) (*p* ≤ .05). The ash content was high in cookies made using wheat flour (WF100%), and the lowest values were seen in those cookies made using MF100% and composite flours (SF75%–MF25% and SF25%–MF75%). The carbohydrate content was high in cookies of composite flour (SF75%–MF25%), and it was low in those made using (SF25%–MF75%). The variations in the chemical composition of cookies made with different flours could be attributed to the differences in the chemical composition of the raw materials. Similarly, previous studies showed differences in the chemical composition of the cookies made using different flours (Giuberti et al., 2018; Jan et al., 2015; Rai et al., 2014; Yildiz & Gocmen, 2021).

**TABLE 4:** Approximate composition, bioactive properties (total phenolic content (TPC) and 2,2‐diphenyl‐1‐picrylhydrazyl (DPPH)), and minerals of developed cookies

Properties	Control (WF 100%)	SF (100%)	MF (100%)	SF25%–MF75%	SF50%–MF50%	SF75%–MF25%
Approximate composition (%)						
Moisture	7.20 ± 0.20b	7.10 ± 0.26b	6.63 ± 0.11c	7.63 ± 0.24a	6.32 ± 0.13 cd	6.20 ± 0.17d
Protein	9.75 ± 0.10c	10.43 ± 0.11b	9.35 ± 0.11d	10.88 ± 0.19a	9.24 ± 0.08d	9.17 ± 0.2d
Fat	28.86 ± 0.27b	27.37 ± 0.25d	28.53 ± 0.24bc	30.27 ± 0.41a	28.30 ± 0.30bc	28.36 ± 0.20c
Ash	1.27 ± 0.05a	1.13 ± 0.05b	0.77 ± 0.01c	0.85 ± 0.01c	1.13 ± 0.05b	0.75 ± 0.01c
Carbohydrate	52.92 ± 0.18d	53.97 ± 0.10c	54.72 ± 0.22bc	50.37 ± 0.42e	55.00 ± 0.72ab	55.52 ± 0.02a
Bioactive properties						
TPC (mg GAE/g)	8.71 ± 2.6f	14.27 ± 2.1d	19.04 ± 1.9b	23.80 ± 1.6a	18.69 ± 3.1c	11.55 ± 2.3e
DPPH (% inhibition)	12.07 ± 1.6e	20.73 ± 1.4d	22.45 ± 1.1c	32.15 ± 1.3a	27.93 ± 1.5b	22.51 ± 1.7c
Minerals (mg/L)						
Potassium (K)	13.13 ± 0.91d	17.91 ± 0.76b	16.48 ± 0.53c	17.76 ± 0.13b	16.94 ± 0.28c	18.35 ± 0.74a
Sodium (Na)	90.18 ± 1.09d	96.53 ± 0.76b	96.23 ± 1.03b	97.07 ± 1.37b	92.99 ± 1.93c	151.8 ± 1.98a
Magnesium (Mg)	13.49 ± 0.09d	17.55 ± 0.42b	15.03 ± 0.71c	17.03 ± 0.37b	17.30 ± 0.12b	18.34 ± 0.91a
Iron (Fe)	7.16 ± 0.83a	<0.5 ± 0.01e	3.03 ± 0.69b	0.79 ± 0.06d	0.82 ± 0.02c	0.84 ± 0.04c
Phosphorus (P)	9.21 ± 1.00d	20.49 ± 0.96a	17.27 ± 0.83c	18.47 ± 0.16b	17.76 ± 0.26c	18.42 ± 0.19b
Zinc (Zn)	<0.5 ± 0.01a	<0.5 ± 0.01a	<0.5 ± 0.01a	<0.5 ± 0.01a	<0.5 ± 0.01a	<0.5 ± 0.01a
Calcium (Ca)	58.14 ± 1.09d	62.76 ± 0.79b	58.61 ± 1.00d	62.32 ± 0.56b	60.24 ± 0.19c	65.18 ± 0.89a

*Note*: Values followed by different letters within each row significantly different (*p* ≤ .05).

Abbreviations: MF, Millet flour; SF, Sorghum flour; WF, Wheat flour.

The bioactive properties (total phenolic content [TPC] and DPPH radical scavenging activity) of cookies indicate that the incorporation of sorghum and millet in the formulations significantly (*p* ≤ .05) enhanced these properties (Table 4). The highest values of TPC and DPPH radical scavenging activity were found in cookies made using the composite flour of SF25%–MF75%, while the least values were seen in cookies made using wheat flour (WF100%) (*p* ≤ .05). This could be attributed to the richness of sorghum and millet with bioactive compounds as well as compound forms during Millard reaction with such melanoidins that possess antioxidant activity. In agreement with our findings, several studies indicated that the incorporation of gluten‐free flours in cookies formulations significantly enhanced the total phenolic contents and the antioxidant activity of the cookies (Giuberti et al., 2018; Hussain et al., 2020; Jan et al., 2015; Yildiz & Gocmen, 2021).

Mineral analysis also showed significant variations among cookie samples (Table 4). The highest (*p* ≤ .05) values of potassium, sodium, magnesium, and calcium were found in cookies made using the composite flour of SF75%–MF25% and the least values of these elements were seen in cookies made using wheat flour (WF100%). The iron content was the highest in cookies made using wheat flour (WF100%), and the least value was found in cookies made using sorghum flour (SF100%) (*p* ≤ .05). The cookies made using SF100% showed the highest values of phosphorus, whereas those made using MF100% showed the least values (*p* ≤ .05). High levels of minerals in cookies made using composite flour of sorghum and millet are attributed to the use of whole flours of these grains, which contain the bran that is known for its richness of nutritional elements. A previous study has shown that magnesium, phosphorus, and potassium were higher in cookies made using sorghum flour than those made using wheat flour (Rao et al., 2018), which is comparable to the findings of the current study.

### 3.4 Sensory attributes of cookies

**FIGURE 2:**
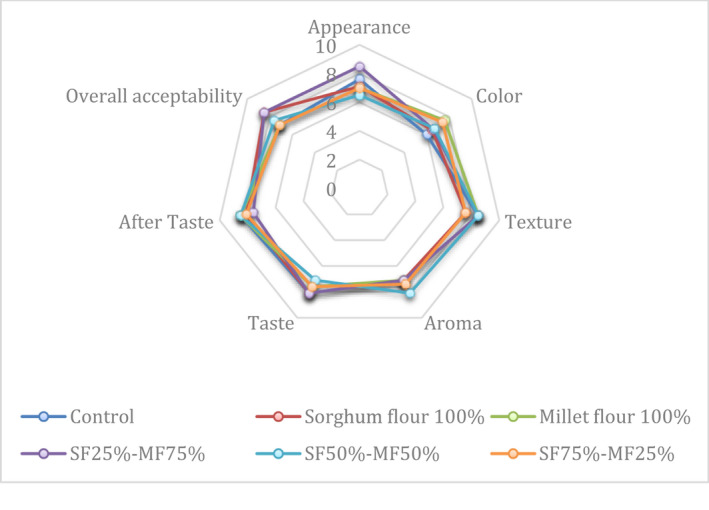
Sensory profile of cookies

The sensory attributes of cookies made using WF, SF, MF, and composite flour of different levels of sorghum and millet flours are depicted in Figure 2. The scores of all sensory attributes of all cookie types were higher than the cutoff score (5), suggesting the acceptance of all cookies by panelists. Generally, there are no significant differences in the sensory attributes among the cookie types. The cookies made using composite flour of SF25%–MF75% outscore others in terms of appearance and overall acceptability, and it shares high scores of texture and taste with other samples indicating the usefulness of this combination (SF25%‐MF75%) for the development of cookies. Similarly, previous studies reported sensory scores to be more than the cutoff score of all sensory attributes of cookies made using gluten‐free flours (Giuberti et al., 2018; Hussain et al., 2020). Another study showed no significant differences in the sensory attributes between the cookies made using gluten‐free flours (Paesani et al., 2020).

## 4 CONCLUSION

This study concludes that cookies can be developed using millet and sorghum flour blends with acceptable physical and nutritional quality. The incorporation of sorghum and millet flour in cookies formulation improved the physicochemical, nutritional, and bioactive properties of the cookies without a major effect on the sensory quality. The developed gluten‐free cookies can be good foods for people suffering from gluten intolerance and those with low income. Based on this study, it is highly recommended that further research should be done to explore gluten‐free cookies from sorghum and millet in nutritional properties and clinical studies are recommended.

## CONFLICT OF INTEREST

No potential conflict of interest was reported on behalf of all authors.

## Data Availability

The data used to support the findings of this study are included within the article.
